# Total femur replacement, indications for the procedure and its complications: a systematic review

**DOI:** 10.1007/s00402-025-05887-9

**Published:** 2025-04-29

**Authors:** Bartłomiej Wilk, Małgorzata Rojek, Julia Gugulska, Paweł Kasprzak, Zofia Wrześniak, Łukasz Pulik, Paweł Łęgosz

**Affiliations:** 1 Medical University of Warsaw, 61 Żwirki i Wigury St, 02-091 Warsaw, Warsaw, Poland; 2 Department of Orthopedics and Traumatology, Medical University of Warsaw, 4 W. H. Lindleya St, 02-005 Warsaw, Warsaw, Poland

**Keywords:** Total femur replacement, Total femur arthroplasty, Indications, Complications

## Abstract

**Introduction:**

Total femur replacement (TFR) is a solution that allows orthopedic surgeons to address the most complicated cases in hip, femur, and knee reconstruction. Complete femur prostheses are used in both oncologic patients with femur tumors that require resection and in cases of complicated prosthetic failure with massive bone defects. TFR is an alternative to hip disarticulation that allows the patient to spare the limb and preserve its function. However, the indications for surgery vary and the procedure has been reported to present a high risk of serious complications.

**Materials and methods:**

We follow the Preferred Reporting Items for Systematic Reviews and Meta-Analysis (PRISMA) guidelines. An electronic database was searched for articles reporting indications, complications, and primary diagnoses prior to TFR. We reviewed study type, number of patients, and complications reported in the study.

**Results:**

15 articles including 651 patients were analyzed. Primary diagnoses before TFR can be divided into two groups: oncological and non-oncological. In some articles, there is no clear demarcation between the indications for the surgery and the primary diagnosis prior to TFR. The most common primary diagnosis in oncological patients was osteosarcoma (48,3%) and in non-oncological patients it was osteoarthritis (42,2%). The most common indications for the procedure were oncological (50,8%), followed by fracture (17,1%) and loosening (8,9%). The most common categories of postoperative complications were infection (32,4%), soft tissue failure (13,9%), and dislocation (12,9%).

**Conclusion:**

Surgeons must be aware of the high risks of complications related to the procedure and should qualify their patients after careful mutual consideration and with a personal approach to potential risks and an overall prognosis. All percussions should be made to avoid periprosthetic infection as main complication. After the surgery any findings indicating possible infection should be investigated to avoid implant failure. High risk of dislocation indicates that more constrained or dual-mobility cups should be considered.

## Introduction

Total femur replacement (TFR) is a solution that allows orthopedic surgeons to address the most complicated cases in hip, femur, and knee reconstruction. The history of TFR began in 1965, when a patient with massive bone loss in the femur, caused by Paget’s disease, was treated for the first time with a TFR prosthesis [1]. The entire femur prostheses are used in both oncologic patients with tumors in the femur that require resection and in cases of complicated prosthetic failure or massive bone loss [2–6]. [2–6]. In addition to this division of patients who undergo TFR, the specific indications for surgery vary and the choice of the procedure is not described as based on any algorithm [2, 3, 7]. Therefore, the treatment strategy may depend on the knowledge and experience of the surgeon. What is more, in some articles, there is no clear demarcation between the indications for the surgery and the primary diagnosis before TFR which may be misleading when interpreting the currently available literature [8, 9].

TFR is an alternative to hip disarticulation allowing to spare the limb and preserve its function instead of amputation. However, the procedure has been reported to present a risk of serious complications. The authors describe various scenarios including periprosthetic infections, dislocations, aseptic loosening of the prosthesis, and many others that often lead to revision surgery or even limb amputation [3, 7]. In some articles, Henderson classification is used to classify complications into 5 types [2, 4, 5, 8–11]. Nevertheless, there is no standard for reporting the outcomes of the procedure.

Nowadays, with increasing life expectancy [12], the population of patients eligible for TFR is expected to grow [13]. This may lead to an increased need for evidence supporting the procedure. Since TFR is not performed frequently, it is not uncommon for studies to have a small sample size, which may be one of the reasons why the results differ [2, 14, 15]. Currently, there are no studies summarizing the most common indications for TFR and its complications.

Therefore, we decided to conduct a systematic review of articles that describe the indications and outcomes of TFR. The review aimed to answer the following questions: 1. What primary diagnoses are the most common prior to TFR? 2. What are the most common indications for TFR? 3. What complications are the most common after TFR?

## Materials and methods

### Search strategy

We searched the PubMed database from its inception (1974) to January 4, 2024. The following search terms were used: (“total femur replacement”) OR (“total femoral replacement”) OR (“total femur prosthesis”) OR (“total femoral prosthesis”) OR (“total femur arthroplasty”) OR (“total femoral arthroplasty”) as filters for studies identification.

### Eligibility criteria

The inclusion criteria for our review were: study type (cohort studies, case control studies, or case series), language (studies published in English), number of patients undergoing total femur replacement (at least 10), and information about complications of the procedure reported in the study.

The exclusion criteria for the review were: study type (case reports, non-peer reviewed publications), number of patients (studies with sample < 10 patients), language (publication in a language other than English), and studies with no complications of TFR reported.

No additional filters on patient age, year of publication, or minimum follow-up were added.

### Selection process and data collection

**Fig. 1:**
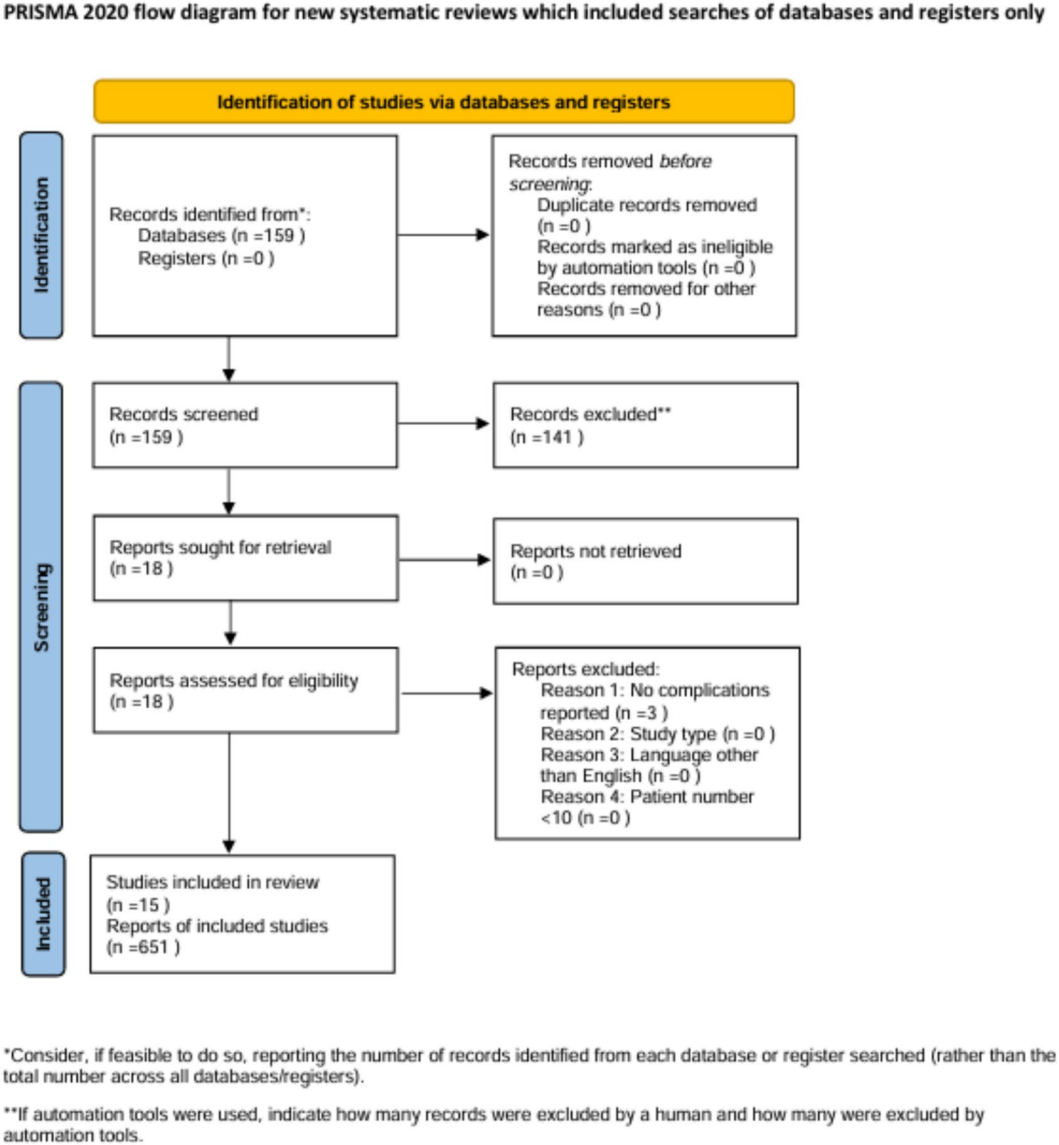
Flow diagram. Preferred Reporting Items for Systematic Reviews and Meta-Analysis (PRISMA) flow diagram with each phase of the data screening process documented

For the data collection process double-screening was performed. The Preferred Reporting Items for Systematic Reviews and Meta-Analysis (PRISMA) guideline was used. Each phase of the data screening process was documented in the PRISMA flow diagram (Fig. 1). There were 3 reviewers. They independently screened the eligibility of the studies. The final decision was made after consultation with other reviewers.

The first selection was based on the titles and abstracts of the studies using our eligibility criteria. The results of the screening were put in the Microsoft (MS) Excel table with information such as titles, first author, and year of publication using the terms'YES'(for studies processed positively) or ‘NO’ (for studies processed negatively following our eligibility criteria).

For studies processed positively, a second screening was made by manuscripts analysis with inclusion and exclusion criteria and were put into the MS Excel table with the reason for inclusion or exclusion. The quality of individual studies and the risk of bias were not assessed since low-evidence studies were included.

The main characteristics of all included studies were displayed in new MS Excel table with variables such as: number of patients (with male to female ratio), primary diagnosis (oncologic and non-oncologic), indication for TFR, mean time of follow-up and mean time of complications appearance after procedure, method of treating the complication (reoperation, antibiotic therapy), number of patients with complication, % of complications, amount of amputations and deaths, mean time from TFR to death, complications type (with number of patients) also using Henderson classification.

The results of the complications reported from included studies were classified by the type of complication into: Implant failure (mechanical failure, structural failure, unspecified), soft tissue failure (disturbed wound healing, hematoma, unspecified), dislocation (hip, unspecified), infection (superficial, deep, chronic, unspecified), aseptic loosening, palsy, instability, arthrofibrosis, subluxation, fracture, tumor progression, amputation, death.

## Results

Preliminary search through PubMed revealed 159 studies. In the titles and abstract selection process, we excluded 141 studies. The remaining 18 studies were assessed for eligibility in full-text review which led to the exclusion of 3 studies with no complications reported. 15 studies were eligible and included in the review. The study selection process was summarized in the flow diagram (Fig. 1).

The studies included in the review varied when it comes to the number of patients, male to female ratio, initial diagnosis (oncological vs. non-oncological patients), mean age of patients, and average follow-up period. The characteristics of the included studies are summarized in the Table 1.

**Table 1:** Characteristics of the included studies

Author (Y)/Study	Patients (n)	Female (n)	Male (n)	Oncological (n)	Non-oncological (n)	Age (mean years)	Average follow-up period
Friesecke C (2005)[6]	100	87	13	4	96	68 years (40–94)	59 months
Sevelda F (2015)[10]	44	22	22	44	0	conventional: 34 years (5–81); expandable: 9 (4–13)	57 months (conventional), 172 months (expandable)
Liu T (2016)[16]	21	8	13	21	0	21.8 years (15–38)	71.2 months
Toepfer A (2016)[4]	18	14	4	0	18	Group A periprosthetic fracture: 77 years (67–90); Group B aseptic loosening: 79 years (70–88)	80 months
Toepfer A (2018)[5]	22	18	4	9	13	Group A malignant musculoskeletal disease: 47 years (36–82); Group B failed revision arthroplasty: 73 years (64–90)	63 months
Sevelda F (2018)[14]	11	8	3	11	0	64 years (41–78)	31 months
Medellin MR (2019)[9]	81	38	43	34	48	43 years (12–86)	10.3 years
Putman S (2019)[17]	29	15	14	0	29	68 years (32–85)	6 years
Graulich T (2019)[18]	22	13	9	6	16	64 years (22–85)	18 months
Muratori F (2019)[8]	32	19	13	23	9	54.2 years (13–82)	60 months
Henderson ER (2020)[11]	166	84	82	155	11	42 years (6–96)	min. 2 years
Christ AB (2020)[15]	16	8	8	0	16	68 years	3.9 years
Adzhar AL (2023)[2]	10	6	4	5	5	20.4 years	17.6 years
Murray J (2023)[7]	37	24	13	0	37	73 years (42–80)	10 years
Mori T (2023)[3]	42	19	23	42	0	46.5 years	37 months

Demographic characteristics of publications included in the review with information about number of patients divided into sex, primary diagnosis (oncological or non-oncological), mean age and average follow-up period

The 15 publications which were found eligible for this review did not report any biases in their individual studies.

### Primary diagnoses

It is important to establish a distinction between direct indication for TFR surgery and patients’ primary diagnosis; TFR is a procedure usually performed as a last resort of limb salvage after previous attempts at treatment and reconstruction have failed.

This distinction has been made because indications for the patient cohort were oftentimes not directly related to the primary diagnoses, and thus including this data more properly demonstrates the full extent of patients’ relevant medical history leading to TFR.

Among 15 considered studies with a combined number of 651 patients 8 studies included the primary diagnoses [2, 3, 6, 10, 14–17]. In these 8 publications, the primary diagnoses can be divided into oncological (143 patients) and non-oncological (130 patients). The most common oncological primary diagnoses were: osteosarcoma (69 patients), chondrosarcoma (15 patients), bone metastasis (14 patients), and Ewing’s sarcoma (13 patients). The most common non-oncological primary diagnoses included osteoarthritis (53 patients), developmental dysplasia of the hip (18 patients), rheumatoid arthritis (17 patients), and unspecified degenerative joint disease (16 patients). The remaining quantitative data of primary diagnoses was included in the Table (Table 2).

**Table 2:** Primary diagnoses

Author (Y)/Study	Oncological							Non-oncological							
	Osteosarcoma	Chondrosarcoma	Bone metastasis	Edwing’s sarcoma	Malignant fibrous histiocytoma	Soft tissue sarcoma	Other	Osteoarthritis	Developmental dysplasia of the hip	Rheumatoid arthritis	Degenerative joint disease	Posttraumatic disease	Fracture	Bechterew disease	Other
Friesecke C (2005)[6]			4					44	18	14		12	2	3	3—tuberculosis (2), infected hip joint (1)
Sevelda F (2015)[10]	21	6		9	3	1	4—spindle cell sarcoma (1), aneurysmal cell cyst (1), malignant Paget disease (1), primitive neuroectodermal tumor (1)								
Liu T (2016)[16]	21														
Sevelda F (2018)[14]			11												
Putman S (2019)[17]							11—tumor resection				16		2		
Christ AB (2020)[15]								9		3		2			2—hip dysplasia (1), post-polio (1)
Adzhar AL (2023)[2]	8						2—giant call tumor (1), chondromyxoid fibroma (1)								
Mori T (2023)[3]	19	9	3	4		6	1—undifferentiated pleomorphic sarcoma of bone								

Primary diagnoses from publications included in the review divided into oncological and non-oncological diagnosis with a given number of patients with specific diagnosis

### Indications

**Fig. 2:**
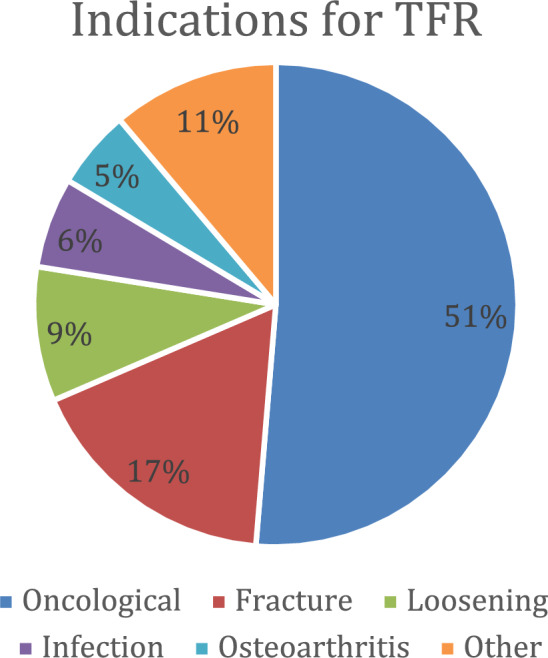
Indications. Circle chart depicting pulled results of the articles included in the systematic review: the most common indications for TFR

Upon analysis, the indications featured in 15 publications included in the review have been divided into 6 categories: infection, loosening, fracture, oncological, osteoarthritis, and others. The most common indications among these categories were oncological (50,8%), followed by fracture (17,1%) and loosening (8,9%). Reported indications from individual studies have been combined in the Table (Table 3) and depicted in the Fig. 2 (Fig. 2).

**Table 3:** Indications

Author (Y)/Study	Infection (n)	Loosening (n)	Fracture (n)	Oncological (n)	Osteoarthritis (n)	Other (n)
Friesecke C (2005)[6]	0	24	41	4	31	complications of previous treated fracture (2)
Sevelda F (2015)[10]	skin lesions (2), iatrogenic contamination (1)	2	after biologic reconstruction (10), pathologic fracture (3), periprosthetic fracture (2)	20	0	0
Liu T (2016)[16]	0	0	0	21	0	0
Toepfer A (2016)[4]	0	7	periprosthetic fracture (11)	0	0	0
Toepfer A (2018)[5]	0	0	0	9	0	failed revision arthroplasty (13)
Sevelda F (2018)[14]	0	0	0	11	0	0
Medellin MR (2019)[9]	osteomyelitis (5)	aseptic loosening (14)	periprosthetic fracture (17)	42	2	implant failure (1)
Putman S (2019)[17]	0	0	2	0	0	degenerative joint disease in the hip and/or knee (16), mechanical failure (11)
Graulich T (2019)[18]	periprosthetic (8)	0	periprosthetic fracture (8)	6	0	0
Muratori F (2019)[8]	0	0	0	23	0	TKR failure (2), failure of total hip revisions (5), revision of TKR and THR (2)
Henderson ER (2020)[11]	0	0	0	155	0	TKR/THR failure (11)
Christ AB (2020)[15]	2	aseptic loosening (5)	periprosthetic fracture (5)	0	0	0
Adzhar AL (2023)[2]	skip lesion (1)	infected loosening + bone loss (1)	1 (with stem loosening)	5	0	total femur allograft flip & pain (1)
Murray J (2023)[7]	20	aseptic loosening (5)	11	0	1	Femoral nonunion after segmental fracture (1)
Mori T (2023)[3]	0	0	0	35	0	relapse after previous tumor resection (5), unplanned surgery that left suspected massive contamination in the femur (2)

Indications for total femur replacement from each publication included in the review, divided into 5 most common groups: infection, loosening, fracture, oncological indications (with information about type of neoplasm), osteoarthritis and others. The values are given as the number of patients

The indications for surgery determine the choice to perform TFR either as a first-line treatment or as a revision implant following the failure of a previous implant. However, due to the scarcity of publications and small patient groups, it is difficult to make an explicit statement upon which strategy is more common.

The oncological patient group remains to be the only one for which TFR is performed as a first-line treatment. The decision is commonly made when no other reconstruction option is considered feasible. In 5 out of the 15 publications, primary TFR constituted the majority of performed procedures: 95% [3], 95% [16], 80% [2], 63% [11], 59% [10], on a total of 199 patients. It must be however pointed out that although Henderson et al. reported 63% of primary TFR, the authors did not clearly mark out the patient group for which this strategy was applied [11].

On the other hand, all of non-oncological and some of oncological patients enrolled in the remaining studies had a history of multiple surgical interventions prior to TFR, constituting either 100% [4–7, 15, 17] or the the majority of the total patient cohorts: 58% [9], 62.5% [8], 72.7% [18], 90.9% [14], together counting 315 patients. Interventions included intramedullary nailing, proximal/distal total femur prosthesis, primary and/or revision TKA or THA, cement spacer with antibiotics, plate osteosynthesis and other. The average number of performed orthopedic procedures was within the range 2.1–4.6 [4, 5, 9, 10, 15, 17]. Friesecke et al. and Putman et al. provided data on prior operations separately at the hip and at the knee, with an average number of 3.2–4.5 surgeries for the former and 0.5–3.6 for the latter [6, 17].

### Complications

Complications reported in the included publications were divided into following categories and subcategories: implant failure (mechanical failure, structural failure, unspecified), soft tissue failure (disturbed wound healing, hematoma, unspecified), dislocation (hip, unspecified), infection (superficial, deep, chronic, unspecified), aseptic loosening, palsy, instability, arthrofibrosis, subluxation, fracture, tumor progression, amputation, death and other. The Henderson Classification was also used to assess complications in 7 out of 15 studies included in the review [2, 4, 5, 8–11].

In the majority of studies the described cohort was a mixed population of oncological and non-oncological patients and the complications were reported without making a distinction for aforementioned groups, thus making it difficult to section off complications for the purpose of this review. Taking that into account, the compiled data featured below is reflective of the reported complications from groups of oncological as well as non-oncological patients.

The combined number of reported complications in the studies included in this review was 395* from the total number of 651 patients. The most common categories of postsurgical complications were: infection (128–32,4%), soft tissue failure (55–13,9%), dislocation (51–12,9%), tumor progression (32–8,1%), and implant failure (25 6,3%). Among the above-mentioned categories, the most common subcategories of complications reported were: unspecified infection (75–19,0%), disturbed wound healing (30 7,6%), hip dislocation (30–7,6%), and periprosthetic infection (23–5,8%).

According to the Henderson Classification, amongst the 7 studies that used it in their assessment with combined 155 reported complications, the most common complications were: Type 4—Periprosthetic infection (59–38,1%), Type 1–Soft tissue failure (46–29,7%) and Type 3—Structural failure (28–18,1%). (Table 4).

**Table 4:** Henderson classification

Author (Y)/Study	Patients (n)	Type 1	Type 2	Type 3	Type 4	Type 5
Sevelda F (2015)[10]	44	14	1	9	7	1
Toepfer A (2016)[4]	18	11	0	2	8	non eligible
Toepfer A (2018)[5]	22	13	0	2	5	0
Medellin MR (2019)[9]	81	1	3	7	15	4
Muratori F (2019)[8]	32	2	0	0	2	1
Henderson ER (2020)[11]	166	5	3	6	21	9
Adzhar AL (2023)[2]	10	0	0	2	1	0

Modes of failure according to the Henderson classification with information about the number of patients from each publication in groups of classification: type 1—soft tissue failure, type 2—aseptic loosening, type 3—structural failures, type 4—infection, type 5—tumor progression

**Fig. 3:**
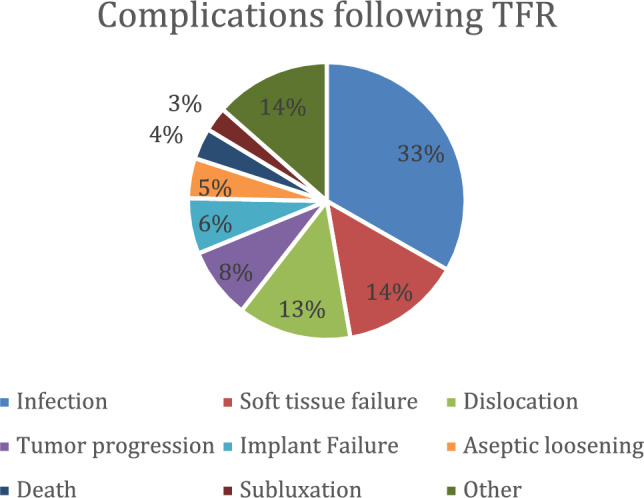
Complications. Circle chart depicting pulled results of the articles included in the systematic review: the most common complications following TFR

Further data about complications reported in individual publications has been summarized in the Table (Table 5) and depicted in the Fig. 3 (Fig. 3).

**Table 5:** Complications

Author (Y)/Study	Implant failure			Soft tissue failure			Dislocation		Infection					Aseptic loosening	Palsy	Instability	Arthrofibrosis	Subluxation	Fracture	Tumor progression	Amputation/Hip disarticulation	Death	Other
	Mechanical failure	Structural failure	Unspecified	Disturbed wound healing	Hematoma	Unspecified	Hip	Unspecified	Superficial	Deep	Chronic	Periprosthetic	Unspecified										
Friesecke C (2005)[6]	3			1	2		6					12			1			1				3	1
Sevelda F (2015)[10]		6		8		14							7	1	5	8				1			6
Liu T (2016)[16]									2	1				3					1	9		9* (due to tumor progression)	
Toepfer A (2016)[4]		3		11				5					8				2						
Toepfer A (2018)[5]	2			6			5			5							2						
Sevelda F (2018)[14]				1 (dehiscence)			1						1		1					1		11	
Medellin MR (2019)[9]							8						15	3				10		4			2
Putman S (2019)[17]		1		3	4		2						8										2
Graulich T (2019)[18]							1					11											
Muratori F (2019)[8]			3						1	2				2						1	3		
Henderson ER (2020)[11]		6				5							21	3						9			
Christ AB (2020)[15]													7	1									
Adzhar AL (2023)[2]			1								1												9
Murray J (2023)[7]								16	5	13				5	1				1				
Mori T (2023)[3]							7						8							7	4		3
Summary	Mechanical failure	Structural failure	Unspecified	Disturbed wound healing	Hematoma	Unspecified	Hip	Unspecified	Superficial	Deep	Chronic	Periprosthetic	Unspecified	Aseptic loosening	Palsy	Instability	Arthrofibrosis	Subluxation	Fracture	Tumor progression	Amputation/Hip disarticulation	Death	Other
	5	16	4	29	6	19	30	21	8	21	1	23	75										
	Implant failure			Soft tissue failure			Dislocation		Infection														
	25			54			51		128					18	8	8	4	11	2	32	7	14	23

Complications after total femur replacement described in included publications with information about number of patients with specific complication divided into implant failure (with a given number of patients with mechanical, structural or unspecified failure), soft tissue failure (with a given number of patients described with disturbed wound healing, hematoma or unspecified failure), dislocation (divided into number of hip dislocations or unspecified), infection (with a given number of patients with superficial, deep, chronic, periprosthetic, or unspecified infection), aseptic loosening, palsy, instability, arthrofibrosis, subluxation, fracture, tumor progression, amputation/hip disarticulation, death and other

Statistical data of reported complications would present differently if studies with a homogenous group of patients were taken into consideration. Analysis of 3 studies with a combined number of 107 exclusively oncological patients [3, 10, 16] suggests that the most common postsurgical complications among them were: tumor progression (17–16,8%), unspecified infection (15–4,9%), unspecified soft tissue failure (14–13,9%) and other (9–8,9%).

Similarly, upon analysis of 2 studies consisting of exclusively non-oncological patient groups with a total of 53 people [7, 15], the most common postsurgical complications among them were: unspecified dislocation (16 32,7%), deep infection (13–26,5%), unspecified infection (7–14,3%) and aseptic loosening (6–12,2%).

However, the number of publications found eligible for this review with exclusively either oncological or non-oncological patient groups was too small to provide substantial data.

### Implant survival over time

In the publications included in the systematic review, there was no standardized method for reporting implant survival over time. The most commonly used metric to describe implant survival was the survival rate at five years. This parameter was reported in five studies (Sevelda et al. [10]: 97% for conventional TFR and 100% for expandable TFR, Toepfer et al. [4]: 56%, Toepfer et al. [5]: 81.8%, Medellin et al. [9]: 81.7%, Muratori et al. [8]: 90%). Three authors reported the revision-free implant survival rate at five years (Sevelda et al. [10]: 48% for conventional TFR and 30% for expandable TFR, Medellin et al. [9]: 71%, Muratori et al. [8]: 87%). Two authors provided data on both the implant survival rate at ten years (Medellin et al. [9]: 72.8%, Muratori et al. [8]: 90%) and the revision-free implant survival rate at ten years (Medellin et al. [9]:63.3%, Muratori et al. [8]: 87%). Furthermore, one study reported the mean time interval between the procedure and implant failure documenting a mean of 41 months [11].

*The given number includes complications from a group of 9 patients from the study by Liu et al. in the category “tumor progression” as well as in the category “death” [16].

## Discussion

The results of this study can be summarized by answering the questions posed in the introduction: 1. What primary diagnoses are the most common prior to TFR? 2. What are the most common indications for TFR? 3. What complications are the most common after TFR? Analysis of the available data concludes that osteosarcoma is by far the most frequent diagnosis prior to TFR in oncological patients (48,3% of oncological patients with reported primary diagnosis) and osteoarthritis in non-oncological patients (40,8% of non-oncological patients with reported primary diagnosis). Indications for TFR were found to be predominantly oncological (50,8%) followed by fracture (17,1%), however, for this categorization the patient cohort was not divided by primary diagnoses into oncological and non-oncological groups. The most common complications that need to be taken into account when considering TFR, according to data available from the included studies, are infection and soft-tissue failure which constituted respectively 32,4% and 13,9% of all combined post-operative complications. Findings from the Henderson Classification were similar pointing to Type 4—Periprosthetic infection (38,1%) as the most common among all reported in the aforementioned classification, closely followed by Type 1—Soft tissue failure (29,7%).

Total femur replacement can deliver satisfactory treatment results, but it is inherently associated with a high risk and should therefore be addressed adequately.

The surgery stands as help of last resort for limb salvage for patients with extensive bone loss, due to oncological or non-oncological indications. The former include extensive femoral tumor involvement, inadequate previous tumor resection, and local recurrence [10, 16], whereas the latter are comprised of periprosthetic fractures, mechanical implant failures, and septic revisions [7, 10]. A significant number of indications are related to infection.

The success rate of TFR is relatively good and the reported revision free prosthesis survival rates reach up to 87% in a 5-year follow-up and 72% in a 10-year follow-up [3, 5, 7–10, 15, 17].

The main factors contributing to the success of the surgery are the possibility of saving from limb amputation, significantly reducing pain, and restoring any function of the limb. Authors report either none [15] or few [6, 10] disarticulations or amputations following a TFR [3, 8, 9, 11]. Pain release is significant, with the majority of VAS scores indicating that patients either feel no pain or suffer from mild pain postoperatively [4, 15]. The function of the limb, classified in the MSTS scoring system, is rated as fair or good, with a score ranging from 13 to 27 [2, 4–6, 8, 9, 16]. Function regain is particularly eminent in patients with periprosthetic fractures, for whom TFR provides stability and allows for immediate weight bearing, in opposition to plate osteosynthesis [6]. Although data clearly shows that the function of the limb is generally low, especially in comparison to the contralateral, unaffected limb, patients generally display small functional demands, and the clinical outcome remains to be superior to that achieved by exarticulation [4, 7, 9, 18]. Pain release, together with partial mobility regain, are the most significant parameters contributing to the patient’s emotional acceptance of the prosthesis [4, 6, 9].

Recent research also reflects on the improvements made both to the surgical technique and the employed prosthesis models, which increased the overall stability and reduced the risk of dislocations. In general, the authors of the majority report a hip dislocations between 0 and 10% [4, 6, 8, 10, 11, 15–17]. Muratori et al. claimed that they managed to reduce the dislocation incidents by about 6.5 times [8]. Toepfer et al. outlined that 67% of their dislocations occurred within the first 2 months after the surgery [5]. Alterations of the implants include the application of dual mobility, large sized heads, tripolar cups, and modular components for version adjustments [4, 8, 9, 11, 14–17]. In terms of the technique, the authors emphasize that better results are achieved when given the possibility to preserve the joint capsule and residual trochanteric bone with tendinous abductor structures, with their consecutive direct attachment to the prosthesis [4, 8, 14, 16]. These results are also consistent with the found correlations between the resection of abductor muscles and hip dislocation [3, 6, 17].

Up to this day, the effectiveness of some reconstruction techniques remains to be undetermined. The doubts pertain to the choice of the hinge at the knee joint and the application of artificial ligament systems at the hip joint. In reference to the hinge design, although many authors suggest that a fixed hinge can provide more stability than a rotating model [15–17], some found no clear superiority of one mechanism over the other [6, 8, 10, 11]. What is more, Medellin et al. had two times more failure rates in fixed hinges in comparison to rotating hinges [9]. In the matter of artificial ligament systems, some authors advise their employment for cases with extensive loss of muscular components, but their effectiveness in dislocation prevention is uncertain and they may increase the risk of infection [3, 8].

Total femur replacement is nevertheless associated with high risks and limitations impeding patient recovery. Due to the development of both the oncological treatment and less invasive reconstructive procedures, surgeons reserve TFR for the most severe cases, which is inherently linked with a high failure rate, reaching up to 72% [4–7, 10, 15, 18]. The most commonly reported reasons for revision are infections, soft tissue failure, wound dehiscence, and chronic pain.

Periprosthetic infections remain to be the biggest concern, constituting up to 44% of reported complications [4–6, 8–11, 15, 17]. Factors of an evidence-based influence on the complication rate are the patient's age, size of both the implant and the wound, the history of prior deep infections as well as prior surgeries, which in the analyzed reports averaged between 1 and 4 operations before TFR [4, 5, 8, 9, 11, 15, 18]. It was found that the reinfection rate is higher than the primary infection rate [4, 6, 11, 17, 18], whereas Medellin et al. reported a 3 times higher risk for infection for those with multiple previous procedures [9]. Factors such as male sex, BMI, diabetes, operative time, radiotherapy, and chemotherapy have a yet undetermined influence on postoperative complications and infections [3, 9, 10, 16, 18]. Current techniques used to minimize the infection risk and efficiently cure ongoing infections are betadine irrigation, debridement, chronic antibiotic suppression, and usage of vacuum [3, 6, 15, 18]. Application of silver coated implants has so far not proved successful, as its concentration significantly decreases after 2 years [8, 9].

Oncological patients display a separate group of risks, which must also be addressed. Namely, both the patient survival and the prosthesis survival are dependent on the tumor type, the risk of recurrence, and metastases. Toepfer et al. argue that the survival of those with extensive tumor disease cannot be improved with either surgical method [5]. Furthermore, research shows a consistent finding that the chances of preserving the limb after recurrence are low and, in most cases, have to end with an amputation due to tumor contamination in the surgical field [9, 11]. Medellin et al. also suggest that their results are reliable only for a mid-term follow-up, as the number of patients who survive over 15 years is too small to provide sufficient data [9]. Lastly, metastatic disease seems to be related to the most complications. Sevelda et al., in their report on 11 patients with metastases, presented weak survival rates, a high rate of deaths and amputations, short-term pain relief, and weak functional outcomes [14]. For this group of patients, palliative care, disarticulation or amputation is advisable.

When it comes to oncological indications for TFR, there is no standard for reporting them among the articles included in our study, which makes it challenging to summarize the data in this systematic review. Some authors present the histological type of the tumor as the indication for the procedure [2, 18]. In two out of three studies with the largest cohorts of oncologic patients included in this review, the authors either report the indications for TFR solely as the histological diagnosis [9], or differentiate primary and revision surgeries, presenting only the revision indications and histological diagnosis without specifying the exact indications for the surgery in primary TFR [11]. Other studies provide more detailed insight into the decision-making process by listing the specific factors that led to TFR in oncologic patients. Sevelda et al. report the indications for the TFR by dividing 44 patients into two groups. In the primary implantation group (26 patients), indications included extensive femoral tumor involvement (77%), pathologic fractures (12%), skip lesions (8%), and inadequate previous tumor resection (4%). In the secondary implantation group (18 patients), indications were fractures after biologic reconstruction with plate fixation (28%), local recurrence (28%), nonunion (11%), cement spacer (11%), implant loosening (11%), periprosthetic fracture (6%), and inadequate previous resection (6%) [10]. Another article created by this author lists only three indications for TFR among 11 oncologic patients: pathologic fracture (73%), recurrence (18%), and osteolysis (9%) [14]. Mori et al. report the following indications for TFR in 42 oncologic patients: large tumor or skip lesion (83%), relapse after previous resection (12%), and massive contamination of the femur after unplanned operation (5%) [3]. As demonstrated, the methods for reporting indications for TFR in oncologic patients vary across studies. A standardized reporting system should be considered in further research to clearly define and compare the exact indications for TFR across studies.

The TFR prosthesis offers a variety of applications for non-oncological indications also. Due to its modular system, the possibility of elongation using interpositional segments, and the fact that the neck-shaft angle, anteversion, as well as the diameter, length, and material of the head can be adjusted, the prosthesis is highly effective in revision arthroplasty following THR or TKR [6, 15]. This solution helps prevent limb amputation or disarticulation [7]. Another benefit is that the TFR prosthesis prevents loss of the limb as the only alternative for those who have had multiple previous surgeries, as well as for those with less compliant soft tissues, such as patients with osteoarthritis [7] or periprosthetic joint infections [19]. The migration of long-stem revision prostheses to the knee joint in infected cases makes the treatment more complex and produces a more stable articulation, which results in a favorable follow-up not only in the case of periprosthetic infections but also periprosthetic fractures [19].

When comparing our work to a systematic review with a similar scope written by Gonzalez et al. we can point out some differences [20]. In our systematic review, we included more detailed information regarding the primary diagnosis and indications for surgery, presenting which ones are the most common. The article presents only a comparison of the outcomes in the oncological and non-oncological groups without revealing specific diagnoses. We provide a detailed description of the most common primary diagnoses (also showing the specific types of neoplasms) and indications, as well as the most frequent complications associated with each indication. The authors show only the outcomes of TFR and the difference in the oncological and non-oncological groups. We included more comprehensive data because we worked not only on studies containing Henderson classifications as the authors did. The authors excluded cases in which previous arthroplasty or reconstruction procedures in bone and/or joints were involved in TFR and we did not. In addition, the inclusion criteria were number of patients undergoing total femur replacement was at least 10 in our review and at least 5 in the article by Gonzalez et al. [20].

Our review has some limitations. A thorough comparative analysis of reported TFR outcomes is rendered difficult due to heterogonous and small patient samples, different tumor stages, responses to therapy, and antibiotic therapies [5, 8, 10, 11, 14, 16–18]. [5, 8, 10, 11, 14, 16–18]. In addition, because of the wide observation timespan recorded in the studies, a significant number of papers correlate results achieved using surgical techniques and implants that evolved over time, further distracting from deriving solid conclusions. Furthermore, all selected studies are retrospective and therefore subject to recall and selection bias, with no control groups. Moreover, the results are reported using a variety of different assessment tools and only some authors used the Henderson classification system [2, 4, 5, 8–11].

## Conclusions

In conclusion, surgeons must be aware of the high risk and limitations associated with the procedure and should qualify their patients after careful mutual consideration and a personal approach to potential risks and an overall prognosis. All precautions should be made to avoid periprosthetic infection as the main complication. After surgery, any findings indicating possible infection should be investigated to avoid implant failure. The high risk of dislocation indicates that more constrained or dual-mobility cups should be considered. TFR is a demanding procedure and should be performed in specialized centers to optimize functional results and minimize complications. Although the number of indications for TFR remains small, a progressively aging population in developed countries, coupled with improvements in oncological treatment, will induce the prevalence of more cases, therefore, it is imperative to continue the analysis of current solutions to find areas that require improvement.

## Data Availability

No datasets were generated or analysed during the current study.
